# Epigenetic Mechanisms in Immune Disease: The Significance of Toll-Like Receptor-Binding Extracellular Vesicle-Encapsulated microRNA

**DOI:** 10.3389/fgene.2020.578335

**Published:** 2020-10-30

**Authors:** Steffi Bosch, Nicholas A. Young, Grégoire Mignot, Jean-Marie Bach

**Affiliations:** ^1^IECM, ONIRIS, INRAE, USC1383, Nantes, France; ^2^Division of Rheumatology and Immunology, Department of Internal Medicine, Wexner Medical Center, Ohio State University, Columbus, OH, United States

**Keywords:** biological relevance, extracellular vesicles, immune activation, epigenetic, innate immunity, Toll-like receptor, miRNA—microRNA

## Why What Is Out There Makes Us Sick?

**Figure 1:**
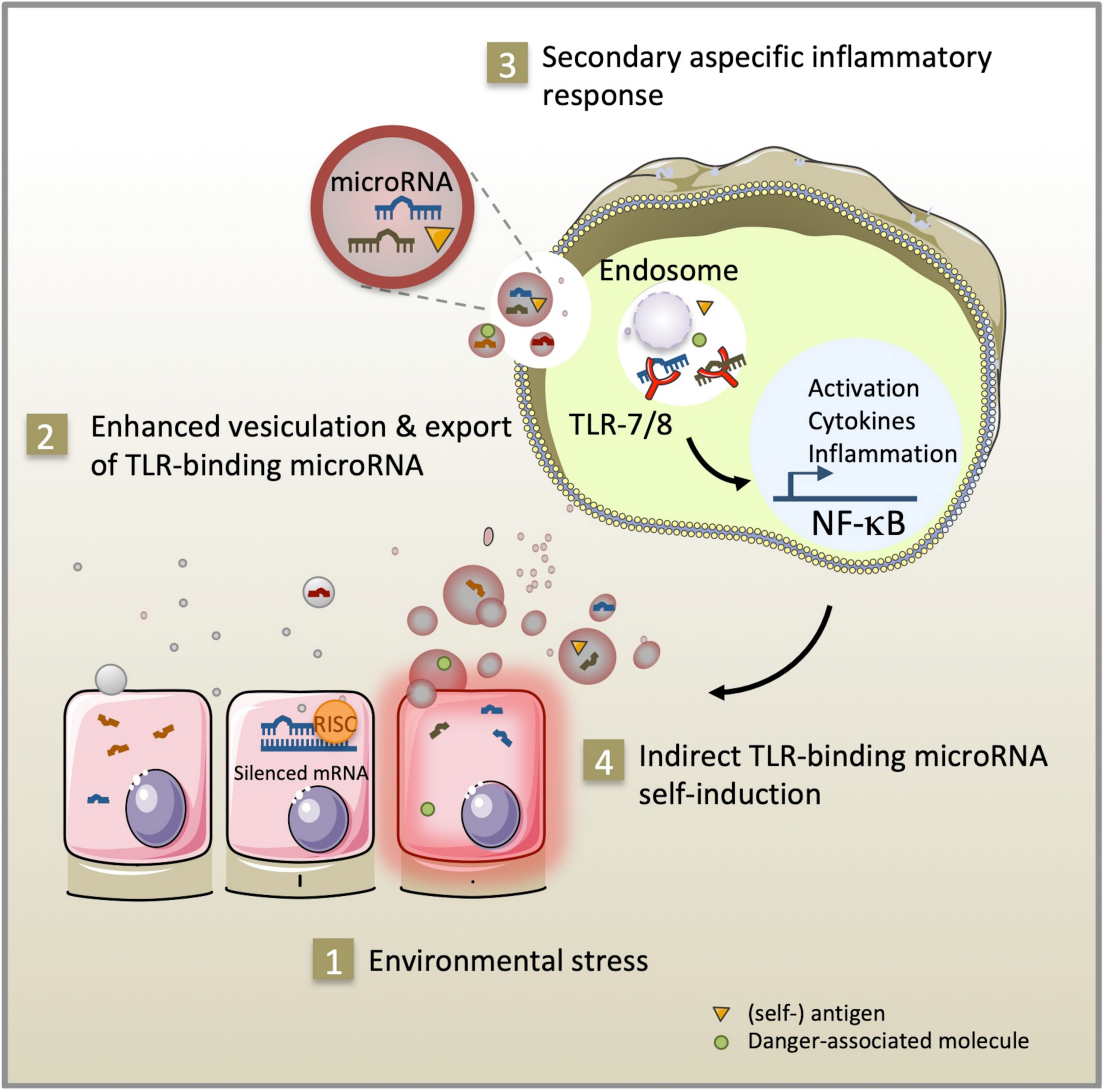
As part of the intercellular endocytic communication pathway, TLR-binding microRNA transmitted via extracellular vesicles serve adaptive and maladaptive stress responses. Environmental stress (1) promotes secretion of extracellular vesicles and microRNA, (self-) antigen and danger-associated molecule release (2). After uptake by innate immune cells, specific GU-rich extracellular vesicle-encapsulated microRNA sequences can stimulate TLR-7/8 signaling in the endosome of recipient cells. Subsequent activation of the NF-κB pathway exacerbates inflammation through cytokine secretion, expression of co-stimulatory molecules (3) and self-induction of TLR-binding microRNA expression and extracellular vesicle secretion (4).

Immune and inflammatory diseases arise from a complex combination of genetic and environmental factors (David et al., 2018; Surace and Hedrich, 2019). MicroRNA are a class of non-coding single-stranded RNA molecules of 19–23 nucleotides in length. In response to environmental triggers, microRNA mediate epigenetic cell fate decisions critical in immune homeostasis by driving cellular activation, polarization, and immunological memory cell development (Mehta and Baltimore, 2016; Curtale et al., 2019). Pattern recognition receptors (PRR) recognize conserved molecular components of pathogens and respond by secreting reactive oxygen species and cytokines that alert the immune system about infection (Medzhitov et al., 1997). They can also interact with various endogenous ligands i.e., lipids, glycans, proteins, and nucleic acids, when released under sterile conditions of cellular stress, tissue injury, and transplantation. As activators of PRR-signaling, endogenous ligands initiate immune cell recruitment and tissue repair. However, sustained PRR-signaling may result in an exacerbated inflammatory response, which can have lethal effects or lead to autoimmunity (reviewed in Yu et al., 2010). In addition to their well-documented canonical function regulating gene expression through RNA interference in the cytoplasm (Bartel, 2004), specific GU-rich microRNA sequences can activate pro-inflammatory signaling pathways by direct interaction with the ribonucleic-acid binding Toll-like receptor 7/8 (TLR-7/8) of innate immunity located in cellular endosomes (Heil et al., 2004). Extracellular vesicles are a heterogeneous population of membrane vesicles naturally secreted by living cells that facilitate intercellular exchanges (Valadi et al., 2007; Raposo and Stoorvogel, 2013). Exported inside extracellular vesicles, Toll-like receptor-binding microRNA released by cells from injured or stressed tissues can reach the endosomal compartment and propagate inflammatory signals in distant recipient cells (Figure 1). The contributions of a dozen of TLR-7/8-binding microRNA (let-7b/c, miR-7a, miR-21, miR-29a/b, miR-34a, miR-122, miR-133a, miR-142, miR-145, miR-146a, miR-208a, and miR-210) to inflammation have been described to date in settings of cancer, sepsis, neurological, autoimmune, and graft-vs.-host diseases (Fabbri et al., 2012; Lehmann et al., 2012; He et al., 2014; Park et al., 2014; Salama et al., 2014; Liu et al., 2015; Yelamanchili et al., 2015; Kim et al., 2016; Coleman et al., 2017; Feng et al., 2017; Ranganathan et al., 2017; Young et al., 2017; Salvi et al., 2018; Xu et al., 2018; Wang et al., 2019). Using confocal microscopy co-localization, co-precipitation, and TLR inhibitors, these studies demonstrate direct binding of these microRNA to TLR-7 in mouse and TLR-8 in human, independently of RNA interference. Furthermore, transgenic TLR-7^−/−^ mice are protected against the degenerative and inflammation-related effects of TLR-binding microRNA (Fabbri et al., 2012; Lehmann et al., 2012; Yelamanchili et al., 2015; Liang et al., 2019). Since their discovery in 2012, the significance of microRNA as endogenous ligands of innate immunity in health and disease is still a matter of debate (Chen et al., 2013; Fabbri et al., 2013; He, X. et al., 2014; Bayraktar et al., 2019). As part of the dynamic continuum of the endocytic intercellular communication pathway, TLR-binding microRNA transported via extracellular vesicles likely serve both adaptive and maladaptive stress responses in cells expressing TLR-7/8.

## Microrna TLR-Binding Activity: An Extracellular Vesicle Phenomenon?

So far, unconventional TLR-binding activity has been observed solely for extracellular microRNA and, out of 14 studies, 11 ascertain transfer in association with extracellular vesicles. The effects of danger-associated molecular patterns depend on their detection, a truism applicable to TLR-7-binding microRNA: they can act as such if and only if they reach the endosomal compartment. Encapsulation within extracellular vesicles constitutes a means for microRNA to enter the endocytic pathway where they may directly engage TLR-7/8 signaling (Mulcahy et al., 2014). In contrast, for RNA-interference activity, internalized microRNA have to escape from the endosome (Montecalvo et al., 2012), a rate-limiting step identified in the delivery of therapeutic short interference RNA (Johannes and Lucchino, 2018) and viral infection (Staring et al., 2018). It is conceivable that TLR-binding microRNA are conducive to exerting RNA interference-mediated effects in donor cells and TLR-binding effects or combinations of both after transfer via extracellular vesicles in recipient immune cells, i.e., major sites of TLR-7/8 expression (Lin et al., 2020; Sun et al., 2020).

The relative proportion of free and particulate microRNA in biofluids still raises controversy, which is in part linked to technical pitfalls in the proper assessment of RNA concentrations in extracellular vesicles and biofluids (Arroyo et al., 2011; Turchinovich et al., 2011; Gallo et al., 2012; Crossland et al., 2016; Jeppesen et al., 2019). While free soluble RNA are short-lived due to high physiological levels of ribonuclease activity, microRNA chaperone protein complexes, or extracellular vesicle microRNA have sufficiently low clearance to support autocrine and paracrine signaling loops (Mitchell et al., 2008). Interaction of extracellular vesicles with patrolling immune cells can further transmit local signals of inflammation to the level of the organism. Useful on one hand for systemic coordination, this transmission can prove detrimental in the case of self-sustaining inflammatory responses. Indeed, let-7b for example, whose production can be enhanced by NF-κb activation (Wang et al., 2012) is also a potent TLR-ligand and thus may enhance its own synthesis, a mechanism perpetuating the vicious circle of inflammation in rheumatoid arthritis (Kim et al., 2016). We have demonstrated previously that liposome-encapsulated miR-21 can induce enhanced extracellular secretion in hematopoietic cells through TLR-7/8 signaling (Chang, 2010; Yang et al., 2015; Young et al., 2017). Similarly, the activation of the type 1 interferon/NF-κb pathway has been shown to induce let-7e, miR-21 and miR-146a expression by a positive amplification loop (Chang, 2010; Yang et al., 2015).

## Quantity Matters

If the body produces endogenous ligands of innate immunity, then how does this influence immune homeostasis? Fabbri and colleagues suggested that it is “the type and amount of information that cells exchange that ultimately affect cancer phenotype” (Fabbri et al., 2013). Indeed, biological active cargo is exported within extracellular vesicles sometimes at higher concentrations than in the donor cells and enhanced vesicle release has been broadly associated with inflammation and degeneration in pathological settings (Valadi et al., 2007; Zomer et al., 2015; Robbins et al., 2016; Young et al., 2017; Giri et al., 2020). As detoxifying “garbage bags” (Vidal, 2019), the enhanced extracellular vesicle outflow is presumably beneficial for the donor cell by permitting material clearance, but might entail deleterious consequences for the organism as a whole. The largely overlapping data reported in biomarker studies have built consensus indicating that measurable changes in circulating microRNA do not directly mirror changes in the diseased tissue, but are indicative of a secondary non-specific inflammatory response (Chen et al., 2008; Witwer, 2015). Presumably not by coincidence, TLR-binding microRNA overexpression is recurrently observed in pathological settings for miR-21, miR-7 and members of the let-7 and miR-29 families opening the way for subsequent polyvalent stimulation of the immune system. Although seemingly a critical factor, the quantitative requirements to modulate functional cellular responses are not well-understood. The biological activity of extracellular vesicle-encapsulated microRNA in recipient cells was first demonstrated in microRNA overexpression reporter experiments *in vitro* or after the transfer of concentrated suspensions of purified vesicles (Kosaka et al., 2010; Montecalvo et al., 2012). However, the number of copies measured per vesicles of a given endogenous microRNA is very low, even for abundant microRNA in extracellular vesicles, which raises questions about the physiological relevance of cell-to-cell microRNA-based communication (Williams et al., 2013; Chevillet et al., 2014). For RNA interference–mediated effects, a threshold of 1,000 copies of microRNA has to be reached in the recipient cells to trigger measurable effects (Brown et al., 2007), which represents the successful delivery of an estimated ≈10^5^ extracellular vesicles (Igaz, 2015). While these concentrations seem realistic for extracellular vesicles released from broadly distributed tissues such as blood, fat, or muscle (Sender et al., 2016), this seems unlikely for less abundant cell types. In contrast, in the attoliter (10^−18^ L) volume of the endosome, a single RNA molecule equates to a 3 μM concentration, which given the micromolar-affinity of the TLR-7 receptor for guanosine-uracil oligomers, might more easily elicit an immune response (Crozat and Beutler, 2004; Zhang et al., 2016). If present, distinct TLR-binding microRNA sequences could synergistically activate TLR-7/8.

## Are All Extracellular Vesicles Equal TLR-7/8 Stimulators?

The absolute quantity and diversity of microRNA exported is highest in large apoptotic bodies and shedding microvesicles. Yet, the majority of evidence on TLR-binding microRNA activity has focused on small ~100 nm vesicles. Similarly, among extracellular vesicles released by serum-starved endothelial cells, only small exosome-like vesicles display immunogenic properties involving the activation of innate PRR by a specific repertoire of non-coding self-RNA (Hardy et al., 2019). In addition to being a consequence of exosome-focused research predominating the field in the past decade, this phenomenon may be explained by evidence of preferential sorting of GU-rich RNA and TLR-binding microRNA into small vesicles in situations of stress (Kouwaki et al., 2016; Fleshner and Crane, 2017; Hardy et al., 2019; Giri et al., 2020; Mensà et al., 2020). Differences in the mechanism and cellular targeting of extracellular vesicle uptake could further influence the impact of TLR-ligand microRNA on recipient cells. Studies on synthetic RNA-containing particles provide evidence that nanometric particles are selectively internalized by plasmacytoid dendritic cells leading to the production of large amounts of interferon-α whereas micrometric particles preferentially induce tumor necrosis factor-α secretion from monocytes (Rettig et al., 2010). The authors infer that, in addition to surface protein expression, nano- or micro-particle size discrimination *per se* allows the immune defense to adapt to viral or bacterial/fungal infection, respectively. In line with this hypothesis, small extracellular vesicles from systemic lupus erythematosus patients and apoptotic lymphoblasts readily stimulate interferon release form plasmacytoid dendritic cells via TLR-signaling (Schiller et al., 2012; Salvi et al., 2018).

In contrast, extracellular vesicles derived from healthy tissue are essentially immune-silent (reviewed in Fleshner and Crane, 2017). For apoptotic bodies, the intrinsic tolerogenic properties rely on the expression of “find” and “eat-me” signals like phosphatidyl-serine that promote the production of anti-inflammatory mediators like the cytokine transforming growth factor-β and the prostaglandin E2 (Fadok et al., 1998; Pujol-Autonell et al., 2013). Equivalent signals may be absent, weak, or masked in microvesicles and exosomes in pathological settings. Indeed, encapsulated inside extracellular vesicles, microRNA are delivered as a bundle, along with many other immune active molecules i.e., lipids (Sagini et al., 2018), cytokines (Fitzgerald et al., 2018), prostaglandins (Lacy et al., 2019), auto-antigens, ATP or danger-associated molecules (Chalmin et al., 2010; Fleshner and Crane, 2017), which have been shown to concentrate in small extracellular vesicles in acute stress responses (Beninson et al., 2014). Evidence from kidney transplant recipients suggests that small exosome-like vesicles released from stressed or injured tissues create a permissive environment promoting the production of autoantibodies against formerly cryptic antigens (Dieudé et al., 2015; Cardinal et al., 2017). In concert with extracellular vesicle-independent co-stimulants, these factors may further shape the outcome of immune responses that rely on the combination of several activation signals.

## Conclusions

As part of the oldest arm of the immune system, TLR developed 1,350 million years ago to adapt to environmental changes by controlling the activation and differentiation of immune cells by epigenetic mechanisms (Nie et al., 2018). Recent drastic alterations in our environment have been linked to an imbalance in immunity and the spread of inflammatory diseases. As catalyst of inflammation, the physiological significance of extracellular vesicle-encapsulated microRNA binding to TLR-7/8 has probably been over-looked. Further experimental evidence is needed to establish the dominant endogenous activator(s) of the inflammatory response. In particular, we lack (i) studies correlating TLR-binding microRNA expression to disease activity (ii) side-by-side comparisons of the dichotomous function of a given microRNA in its soluble form or encapsulated within specific subpopulations of extracellular vesicles, and (iii) evaluation of extracellular vesicle self-antigen modulation of (auto-) immune responses. The use of animal models should be valuable to further explore thresholds of physiological consequences of TLR-7/8 microRNA activation and systemic interactions in an integrated fashion, *in vivo*. Ultimately, new medication antagonizing TLR-binding microRNA may present an opportunity to prevent excessive inflammatory responses.

## Author Contributions

All authors conceptualized, wrote, edited, and approved the manuscript. SB designed the figure.

## Conflict of Interest

The authors declare that the research was conducted in the absence of any commercial or financial relationships that could be construed as a potential conflict of interest. The reviewer FC declared a shared affiliation, with no collaboration, with one of the authors, NY, to the handling editor at the time of the review.
